# Amino acid substitutions in PBP3 in *Haemophilus influenzae* strains, their phenotypic detection and impact on resistance to β-lactams

**DOI:** 10.1093/jac/dkaf023

**Published:** 2025-02-03

**Authors:** Vladislav Jakubu, Marketa Cechova, Martin Musilek, Lucia Malisova, Barbora Zapletalova, Helena Zemlickova

**Affiliations:** National Reference Laboratory for Antibiotics, Centre for Epidemiology and Microbiology, National Institute of Public Health, Srobarova 49/48, 100 00 Prague 10, Prague, The Czech Republic; Department of Microbiology, 3rd Faculty of Medicine, Kralovske Vinohrady University Hospital and National Institute of Public Health, Charles University, Ruska 87, 100 00 Prague 10, Prague, The Czech Republic; National Reference Laboratory for Antibiotics, Centre for Epidemiology and Microbiology, National Institute of Public Health, Srobarova 49/48, 100 00 Prague 10, Prague, The Czech Republic; National Reference Laboratory for Meningococcal Infections, Centre for Epidemiology and Microbiology, National Institute of Public Health, Srobarova 49/48, 100 00 Prague 10, Prague, The Czech Republic; National Reference Laboratory for Antibiotics, Centre for Epidemiology and Microbiology, National Institute of Public Health, Srobarova 49/48, 100 00 Prague 10, Prague, The Czech Republic; Department of Microbiology, 3rd Faculty of Medicine, Kralovske Vinohrady University Hospital and National Institute of Public Health, Charles University, Ruska 87, 100 00 Prague 10, Prague, The Czech Republic; National Reference Laboratory for Antibiotics, Centre for Epidemiology and Microbiology, National Institute of Public Health, Srobarova 49/48, 100 00 Prague 10, Prague, The Czech Republic; National Reference Laboratory for Antibiotics, Centre for Epidemiology and Microbiology, National Institute of Public Health, Srobarova 49/48, 100 00 Prague 10, Prague, The Czech Republic; Department of Microbiology, 3rd Faculty of Medicine, Kralovske Vinohrady University Hospital and National Institute of Public Health, Charles University, Ruska 87, 100 00 Prague 10, Prague, The Czech Republic

## Abstract

**Background:**

Data from surveillance on antibiotic resistance have shown an increasing prevalence of non-enzymatic resistance (β-lactamase-negative ampicillin-resistant) to β-lactam antibiotics among *H. influenzae* strains in the Czech Republic. Aminopenicillins are recommended agents for non-invasive *Haemophilus influenzae* infections. The phenomenon of non-enzymatic resistance to β-lactams is complicated by the fact that the phenotypic detection of PBP3 with specific amino acid substitutions (rPBP3) is challenging, since rPBP3 isolates have repeatedly been demonstrated to be split by the epidemiological cut-off values (ECOFF) for aminopenicillins defined by EUCAST.

**Objectives:**

We sought to determine whether the penicillin disc has sufficient detection ability to predict the non-enzymatic mechanism; whether other antibiotics can be used for detection; and what is the agreement between the broth microdilution and disc diffusion methods.

**Methods:**

We undertook susceptibility testing of selected antibiotics according to EUCAST of 153 rPBP3 strains, and sequencing of the *ftsI* gene to determination amino acid substitutions.

**Results:**

For a selected set of rPBP strains: (i) the detection capability for penicillin, ampicillin, cefuroxime and amoxicillin/clavulanate was found to be 91.5%, 94.4%, 89.5% and 70.6%, respectively; (ii) the categorical agreement between the disc diffusion method and the MIC for ampicillin and cefuroxime was 71.1% and 83.8%, respectively.

**Conclusions:**

We observed better recognition of rPBP3 strains by the ampicillin disc than by the penicillin disc. There is frequently a discrepancy in the interpretation of susceptibility results between the methods used.

## Introduction

*Haemophilus influenzae* is a common inhabitant of the upper respiratory tract in humans and is responsible for a wide range of infections. These infections encompass a spectrum of conditions, ranging from relatively benign upper respiratory tract diseases such as acute sinusitis and acute otitis media to life-threatening invasive infections, including meningitis, epiglottitis and bacteraemia.^1,2^

Invasive infections caused by *H. influenzae* are most commonly associated with encapsulated serotype b (Hib). However, the introduction of a Hib vaccine has significantly reduced the overall incidence of Hib invasive infections. Recent reports suggest that most invasive disease is attributed to non-typable *H. influenzae* (NTHi).^3^ The incidence of *H. influenzae* infections in Europe has been reported as 0.8 per 100 000.^3^ In the Czech Republic, following the introduction of Hib vaccination, the incidence of this serotype decreased from 1.1/100 000 inhabitants to 0.19/100 000 inhabitants in 2020. Since 2009, NTHi has been the most common agent of invasive diseases.^4^

Aminopenicillins are recommended for the treatment of non-invasive infections caused by *H. influenzae*.^5^ The first mechanism of resistance discovered was the production of the β-lactamase TEM-1.^6,7^

In 1986, an ampicillin-resistant strain without β-lactamase production was documented.^8^ Since that time, there has been a growing number of cases of ampicillin-resistant strains that do not produce β-lactamase. This mechanism of β-lactam resistance has been found to result from an alteration in the transpeptidase region of PBP3 due to a missense mutation in the *ftsI* gene.^9,10^ β-Lactamase-negative isolates with phenotypic resistance are referred to as BLNAR (β-lactamase-negative ampicillin-resistant). The key mutations lead to amino acid substitutions N526K or R517H,^6,10–12^ which have been confirmed to reduce β-lactam binding to PBP3. Strains exhibiting these substitutions are designated as rPBP3 strains.^13^ The N526K substitution is the most prevalent in rPBP3 strains in Europe and, for example, in Japan.^13–23^

The observation of the occurrence of BLNAR strains commenced in earnest after 2000, when the first countries initiated state-level surveillance of the phenomenon. Over the subsequent 20 years, a considerable quantity of data from national surveillance systems in both European and non-European countries has been collected.^13–23^

Presented data frequently originate from different methodologies (e.g. CLSI and EUCAST),^24^ which can complicate the comparison process. Nevertheless, we concur that the identification of rPBP3 strains by β-lactam susceptibility tests is challenging due to the fact that the values obtained for these strains frequently overlap with those obtained for testing WT ones. The development of breakpoint settings for aminopenicillins also affects the comparison of results. In EUCAST, the clinical breakpoints for susceptibility categories have changed over time for both the disc diffusion and MIC methods.

Our observations indicate that issues with overlapping values and the difficulty of determining the rPBP3 phenomenon persist even when the current breakpoints for the resistant category are employed (EUCAST: ampicillin MIC >1 mg/L; disc <18 mm).^25^ The authors report an initial MIC of ampicillin for rPBP3 strains of 0.5 mg/L, with a maximum of 1 mg/L for the number of strains inhibited,^22,26,27^ which overlaps with the WT distribution.^28,29^ Standard methods therefore have limitations for correct identification of these strains.

Discrepancies between results from the publications mentioned above can be partially attributed to the methodology used, especially differences in media (Haemophilus Test Medium agar versus Mueller Hinton Fastidious agar), and batch variability of media and antibiotic discs observed between manufacturers.^30–32^

The occurrence of mutations in the *ftsI* gene at new sites gives rise to novel combinations of amino acid substitutions.^17,33^ This may also influence the outcomes of β-lactam susceptibility testing.

Additional amino acid substitutions can occur at more than 20 different positions and their prevalence and contribution to resistance levels remain unclear.^17^ Only substitutions that are in close spatial proximity to the conserved motifs in the 3D structure are predicted to lead to an increase in MIC.^34^  *H. influenzae* strains have three functionally critical and highly conserved amino acids at positions 327 (S327-T-V motif, serine-threonine-valine), 379 (S379-S-N motif, serine-serine-asparagine) and 512 (K512-T-G motif, lysine-threonine-glycine) of the PBP3 protein primary structure.^6,10,12^

It seems that alterations in the other PBPs do not contribute to the resistance of *H. influenzae* to β-lactams.^35^ Other described resistance mechanisms, such as overexpression of the efflux pump AcrAB,^35^ caused by mutations in the regulatory gene *acrR*, or changes in porin density, occur very sporadically.

Our work was prompted by the results of regular surveillance of antibiotic resistance in *H. influenzae* causing respiratory tract infections in the Czech Republic, which showed an increasing trend of strains with non-enzymatic resistance to β-lactam antibiotics. Since 2010, there has been an increase in BLNAR prevalence, from 0.8% in 2010 up to 15% in 2020 and 6.7% in 2023 (the reduction in years 2021–22 was influenced by the COVID-19 pandemic).^36^

The aforementioned publications demonstrate that penicillin disc 1 U is the most effective and sensitive test for detection of β-lactam resistance mechanisms, which is in accordance with the EUCAST algorithm.^13,17,22,26,37^ Given that a significant number of clinical laboratories employ the disc diffusion method for susceptibility testing, it is pertinent to enquire whether the penicillin disc remains the most reliable predictor of the rPBP3 mechanism, even in the context of a changing and evolving population of rPBP strains.

Furthermore, we sought to ascertain the efficacy of ampicillin, amoxicillin/clavulanate and cefuroxime discs in detecting rPBP strains. In addition, we were also interested in the categorical agreement between broth microdilution and disc diffusion methods. Finally, we attempted to determine whether all combinations of amino acid substitutions affected the resistance test results in the same way.

## Materials and methods

### Strains

As part of the national resistance surveillance, in 2020 to 2023 clinical laboratories were encouraged to send *H. influenzae* strains with resistance to any β-lactam antibiotic to the National reference laboratory for antibiotics (NRL ATB) for further analysis. Thus, we obtained a preselected population of *H. influenzae* strains. All submitted strains were subjected to genotypic detection of altered PBP3, and all demonstrated the presence of this alteration. A total of 153 strains were collected (2020: *n* = 34 strains; 2021: *n* = 11; 2022: *n* = 34; 2023: *n* = 74). Strain numbers in 2020–2021 were affected by the COVID-19 pandemic. Of the 153 strains tested, 11 were found to be positive for β-lactamase, the remaining 142 were negative. The strains were sourced from 37 laboratories, distributed across all 14 administrative regions of the Czech Republic. Strains were isolated from blood (*n* = 20), lower respiratory tract (*n* = 76), upper respiratory tract (*n* = 36), vaginal swab (*n* = 8), ear (*n* = 7), skin swab (*n* = 2), bile (*n* = 1), eye (*n* = 1) and unknown (*n* = 2).

In the NRL ATB the strains were inoculated on Levinthal agar (Oxoid, the Czech Republic) and incubated overnight at 35 ± 1°C and 5% CO_2_. The identification was performed using MALDI-TOF MS (Microflex, Bruker Daltonics, Germany).

### Susceptibility testing to β-lactams

The NRL ATB tested the susceptibility to penicillin (1 U), cefuroxime (30 µg), ampicillin (2 µg) and amoxicillin/clavulanic acid (2/1 µg) using the disc diffusion method. The MICs of ampicillin, cefuroxime and cefotaxime were determined by the broth microdilution method (BMD). Both methods and interpretation of susceptibility testing results were conducted in accordance with the EUCAST guidelines, version 13.0.^25^ Breakpoints for resistance were set as follows: penicillin screen (disc <12 mm), ampicillin (disc <18 mm, MIC >1 mg/L), cefuroxime (<25 mm,  >2 mg/L), amoxicillin/clavulanic acid (<15 mm) and cefotaxime (>0.125 mg/L). *H. influenzae* strains ATCC 49766 and ATCC 49247 were used as quality controls.

### PCR detection of β-lactamases

β-Lactamase production was examined by the nitrocefin method.^38^ Verification of β-lactamase production was performed by PCR detection of *bla*_TEM-1_ and *bla*_ROB-1_.^39^  *H. influenzae* strains CIP 107112 and CIP 104278 were used as positive controls for the detection of the *bla*_TEM-1_ and *bla*_ROB-1_ genes, respectively.

### Detection of mutations in the ftsI gene and comparative analysis

All strains were sequenced in the 977–1597 bp region of the *ftsI* gene (621 bp fragment) to verify the presence of mutations in the PBP3 transpeptidase region according to the protocol from PubMLST.org.^40^ The nucleotide sequence was converted into an amino acid sequence in Bionumerics 7.6.2 (Applied Maths, Ghent, East Flanders, Belgium) and compared with the amino acid sequence (positions 326–532) of the non-mutated reference strain *H. influenzae* ATCC 51907 (GenBank accession number L42023).^40^

### Statistical analysis

Categorical agreement was calculated as the interpretative agreement between the susceptibility results of the two methods used. To further elucidate the degree of agreement, the kappa statistic was employed. The kappa statistic is a measure of the degree of agreement between two raters when classifying items into predefined categories. In contrast to the simple percentage agreement, the kappa statistic allows for the potential of random agreement to be taken into account. Very major error (VME) and major error (ME) values were also determined. The VME value was calculated based on the difference between the disc and MIC methods, where for one strain the disc results were susceptible and the MIC showed resistance. For the calculation of the ME value, the reverse was true, where for one strain the disc results were resistant and the MIC susceptible.

## Results

### Results of susceptibility testing of β-lactam antibiotics

Of the 153 strains tested, 11 were found to be positive for β-lactamase. The overall prevalence of antibiotic resistance among the strains tested was as follows: 141 strains (92.2%) exhibited resistance to penicillin, 146 (95.4%) to ampicillin, 108 (70.6%) to amoxicillin/clavulanate, 141 (92.2%) to cefuroxime and 34 (22.2%) to cefotaxime. These findings were observed regardless of test method, and data from β-lactamase-producing/non-producing strains were not distinguished. The only β-lactamase detected in the β-lactamase-positive strains was TEM-1, whereas ROB-1 was not detected in a single case.

### Amino acid substitutions

**Figure 1.**
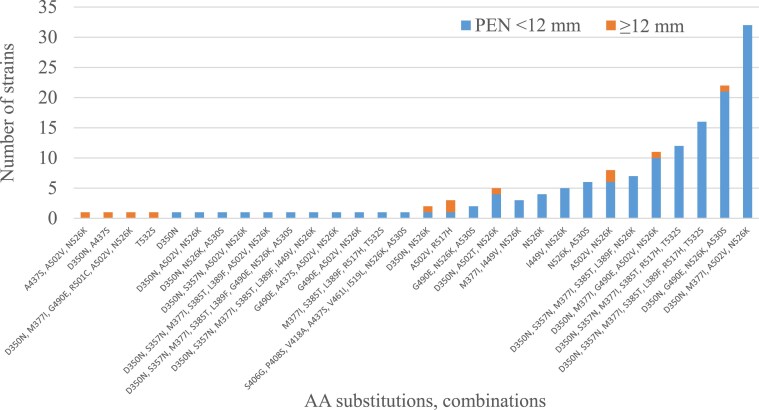
Frequency of amino acid combinations of substitutions and the proportion of positive (PEN <12 mm)/negative (PEN ≥12 mm) strains according to the EUCAST screening algorithm. Positive means a match between the EUCAST screening algorithm (penicillin inhibition zone <12 mm) and the finding of amino acid substitutions in PBP3. PEN, penicillin.

Twenty different amino acid substitutions were identified, occurring individually (in only six strains) or in combinations of two to eight amino acid substitutions. The following amino acid substitutions were found: D350N, S357N, M377I, S385T, L389F, S406G, P408S, V418A, A437S, I449V, V461I, G490E, R501C, A502V, A502T, I519L, R517H, N526K, A530S and T532S. The combinations (*n* = 30) are listed in Figure 1 and Table S1 (available as Supplementary data at *JAC* Online). The most frequently observed combination of substitutions in β-lactamase-positive strains was D350N, S357N, M377I, S385T, L389F, R517H, T532S, occurring in seven strains. The most prevalent combination of substitutions observed in β-lactamase-negative strains was D350N, M377I, A502V, N526K.

All combinations of substitutions in β-lactamase-negative strains can be observed in Figure 1, which shows the frequencies of amino acid substitution combinations and the proportion of positive/negative strains according to the EUCAST penicillin screening algorithm. The N526K base substitution is present in 118 strains in many different combinations, but is present alone in only four strains. The R517H substitution was found in 32 strains. Only three strains did not contain any of these two basic AA substitutions and thus do not meet the definition of rPBP3.

### Phenotypic detection of rPBP and correlation of zone-MIC diameter

The analysis of individual antibiotics and testing methods on the selected set of strains demonstrated that the disc diffusion method with ampicillin yielded the most effective results for the detection of mutant strains (in 94.4%) in the set of measurements. A comparison of the ability to detect strains with mutations using penicillin, ampicillin, amoxicillin/clavulanate and cefuroxime is shown in Table 1.

**Table 1.** Detection ability of rPBP3 detection using the EUCAST 1 U penicillin screening disc and the ampicillin, amoxicillin/clavulanate and cefuroxime discs on the selected set of strains

Antibiotics	rPBP3 detection by disc diffusion method		rPBP3 detection by MIC method		Categorical agreement^b^, %	VME, %	ME, %
	*n*	%	*n*	%			
Penicillin^a^	130	91.5	NA	NA			
Ampicillin^a^	134	94.4	95	66.9	71.1	0.7	28.2
Amoxicillin/clavulanate	108	70.6	NT	NT			
Cefuroxime	137	89.5	117	84.8	83.8	2.2	11.6
Penicillin disc/ampicillin MIC					72.2	1.4	25.4

BMD, broth microdilution method; ME, major error, i.e. resistant by disc diffusion and susceptible by BMD MIC; NA, not available; NT, not tested; VME, very major error, i.e. susceptible by disc diffusion and resistant by BMD MIC.

^a^Penicillin and ampicillin values calculated only from β-lactamase-negative strains (*n* = 142); amoxicillin/clavulanate and cefuroxime from all 153 strains.

^b^Categorical agreement is reported as the percentage of interpretative agreement between the MIC and disc diffusion methods.

In comparison with the disc diffusion method, the MIC assay of ampicillin and cefuroxime proved to be the least suitable for the detection of our set of rPBP3 strains (Table 1). In order to assess the agreement of the two examination methods, the categorical agreement of the susceptibility results and the values for ME and VME were also calculated for our set of strains. The most important ME and VME values between penicillin disc and ampicillin MIC were calculated to be 25.4% and 1.4%, respectively. Table 1 shows the results for all these parameters, and Table S1 shows them for each amino acid substitution combination.

**Figure 2.**
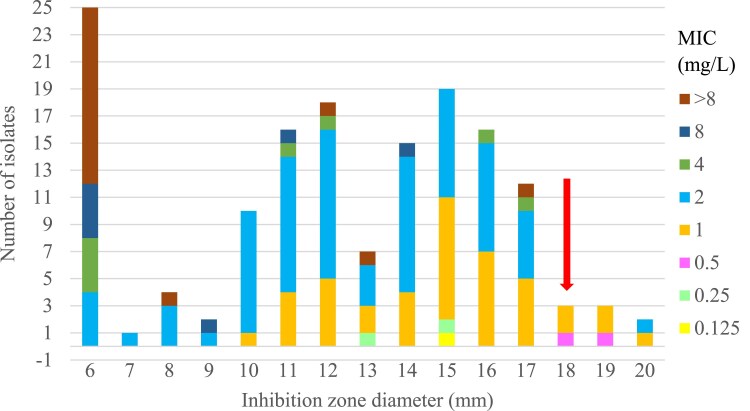
The correlation of ampicillin disc 2 µg and ampicillin MIC: 142 correlates of zone-MIC diameter for β-lactamase-negative *H. influenzae*. The value of the ampicillin breakpoint of disc diffusion for susceptible strains (≥18 mm) is indicated by the arrow. The MIC breakpoint for ampicillin-susceptible strains is ≤1 mg/L.

Figure 2 illustrates a considerable range of both MIC and inhibition zones in our set of strains. It also demonstrates that strains susceptible according to MIC are present at zones below the breakpoint for disc diffusion. A total of 42 strains exhibited an ampicillin MIC of 1 mg/L, indicating susceptibility. However, 37 of these strains were classified as resistant based on disc diffusion.

**Figure 3.**
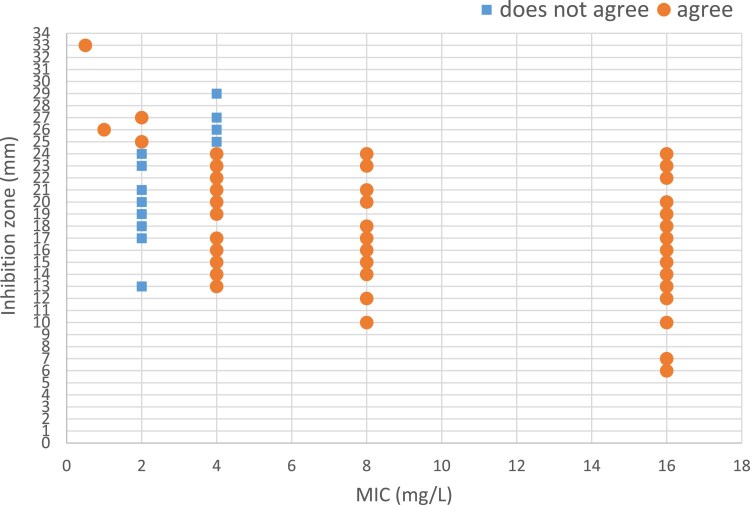
Interpretative agreement (correlation) between MIC and disc diffusion methods for cefuroxime and 138 *H. influenzae* strains. The value of the breakpoint of disc diffusion for resistant strains is <25 mm; the MIC breakpoint for resistant strains is >2 mg/L.

A notable degree of variability was observed in the inhibition zone values in relation to the MIC of cefuroxime. For instance, 15 distinct inhibition zone values were associated with the MIC of 16 mg/L. The correlation between MIC and disc diffusion for cefuroxime shows that the lowest agreement can be found for MIC 2 mg/L and 4 mg/L. From MIC >4 mg/L onwards, there is a perfect agreement between MIC and diffusion (see Figure 3).

**Figure 4.**
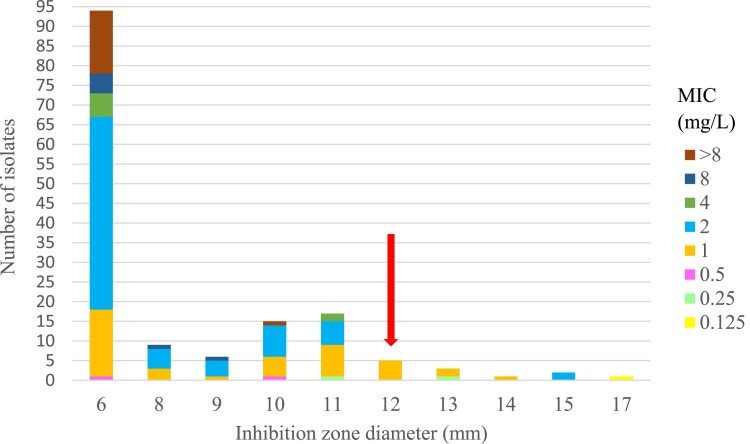
The correlation of penicillin disc 1 U and ampicillin MIC: 142 correlates of zone-MIC diameter for β-lactamase-negative *H. influenzae*. The screening value of penicillin disc diffusion for susceptible strains (≥12 mm) is indicated by the arrow; the MIC breakpoint of ampicillin for susceptible strains is ≤1 mg/L.

Figure 4 is analogous to Figure 2, but illustrates the correlation between the penicillin 1 U disc and MIC of ampicillin. A total of 37 strains exhibiting susceptibility to ampicillin by MIC were found to be below the screening threshold for the penicillin disc.

The kappa statistic values for disc/MIC agreement for ampicillin and cefuroxime were 0.18 and 0.32, respectively. For the agreement between penicillin disc and ampicillin MIC, a value of 0.24 was calculated.

Table S1 contains the following results for a selected population of rPBP strains: categorical agreement (agreement in percentage between MIC and disc diffusion methods); detection value for rPBP3 (how much the combination of the method and the antibiotic is able to detect rPBP3 strains, as percentage); VME (i.e. susceptible by disc diffusion and resistant by broth microdilution MIC, as percentage), ME (i.e. resistant by disc diffusion and susceptible by broth microdilution MIC, as percentage) of all detected amino acid combinations and MIC values of ampicillin and cefuroxime. For penicillin, only the detection limit for rPBP is given.

## Discussion

Data from surveillance of antibiotic resistance in the Czech Republic have shown a growing trend in *H. influenzae* strains with non-enzymatic resistance to β-lactam antibiotics.^36^ One of the aims of this study was therefore to investigate the ability of a panel of β-lactam antibiotics to identify mutations in PBP3 in *H. influen*zae strains and to evaluate the efficacy of various diagnostic techniques for their identification. This could potentially provide guidance to clinical laboratories on how to enhance the ability to detect rPBP3. The MIC method for ampicillin and cefuroxime and the disc diffusion method for penicillin, ampicillin, amoxicillin/clavulanate and cefuroxime were used to detect rPBP3. The antibiotics in question were selected based on the recommendations of the EUCAST for the screening of rPBP3 strains (penicillin) and the use of these antibiotics in clinical practice in the Czech Republic. To achieve the above goals, we used a selection of strains that all contained amino acid substitutions in PBP3 and thus the results cannot be generalized to the entire *H. influenzae* population.

Previous studies on the phenotypic detection of rPBP3 strains have identified two distinct phenomena. A discrepancy between the MIC and the disc diffusion method with susceptibility recorded for discs and resistance for MIC (11% to 28%) was observed in the strains in several studies.^22,26,27,41^ Aguirre-Quiñonero *et al.*^37^ reported the opposite problem, with ampicillin susceptibility for the MIC and resistance for the disc method. The findings of our study are more closely aligned with those presented by Aguirre-Quiñonero *et al*.

Regarding assessment of the detection sensitivity of the penicillin disc in accordance with the EUCAST algorithm, research findings indicate values exceeding 90%. Furthermore, it is asserted that penicillin represents the most efficacious detector of rPBP3. These reports include Aguirre-Quiñonero *et al.*^37^ (93%), Skaare *et al.*^13^ (95.5%), Jakubu *et al.*^17^ (95.7%) and Søndergaard and Nørskov-Lauritsen^22^ (96%). The sensitivity scores in the aforementioned articles are essentially very similar, and the differences can be explained in terms of the technical design of the disc diffusion test and the manufacturer of the agar and antibiotic discs used. In our study, targeting only strains with amino acid substitutions in PBP3, we found a slightly lower value for penicillin (91.5%). Our data set indicates that the ampicillin disc may be more effective for rPBP detection (94.4%). Although the frequency of VMEs for ampicillin did not exceed the 1.5% threshold, the overall level of MEs (28.2%) should have precluded the use of ampicillin disc for rPBP3 detection (threshold 3%).^42^ A similar result was observed when comparing the penicillin disc (1 U) and MIC of ampicillin, where the calculated VME and ME values were 1.4% and 25.4%, respectively. Cefuroxime has the worst VME but the lowest ME of the antibiotics studied. We also focused on establishing agreement between the test methods. The degree of agreement between the disc and BMD methods was found to range from 71% to 84%. However, upon applying the kappa statistic to the data set, a significant decline in the level of interpretative agreement was observed, according to published criteria.^43^ Thus, after kappa was applied, a maximum interpretative agreement of 4% was observed for ampicillin, whereas maximum agreement was 15% for cefuroxime and penicillin discs when compared with the ampicillin MIC.

It should be noted that even with MIC there are differences in the ability to detect modified PBP3. The MIC of ampicillin and the MIC of cefuroxime are inadequate for the recognition of modified PBP3, exhibiting low detection ability of 66.7% and 84.8%, respectively. For most strains resistant to ampicillin by disc diffusion and susceptible by MIC, a MIC value of 1 mg/L was measured; 1 mg/L is the breakpoint value for susceptible/resistant strains. It is evident that the observed values may be subject to variation due to the technical design of the method. In accordance with the standard, a tolerance of  ±1 dilution is generally acceptable in the context of interpreting MIC results,^44^ so the actual value is likely to be between 0.5 and 2 mg/L. Consequently, with the EUCAST breakpoint set at 1 mg/L, there will persist a discrepancy in categorization between disc diffusion and MIC. However, in clinical practice, it is important to ascertain whether the antibiotic concentrations achieved in the blood are sufficient to eliminate rPBP3 strains. According to MIC testing, it appears that for at least some rPBP3 strains, a sufficiently high concentration of ampicillin is achieved in the blood to ensure their inhibition. It would be useful to use clinical trials to determine whether ampicillin treatment actually has clinical outcome in rPBP3 strains with an ampicillin MIC up to 1 mg/L. It is also important to consider the possibility that an rPBP3 strain that is susceptible to ampicillin according to MIC may develop into a resistant strain. Despite the paucity of case reports or articles focusing on the treatment of rPBP strains, Qureshi *et al.*^45^ describe a case of BLNAR strain development in the treatment of meningitis caused by *H. influenzae* type b. In the case described, the strain was susceptible to ceftriaxone at the start of treatment and after 7 days at a dose of 200 mg/kg/day, resistance to third-generation cephalosporins with preserved susceptibility to meropenem was detected. Furthermore, an *in vitro* study has demonstrated that new mutations in the *ftsI* gene can occur in *H. influenzae* strains (WT or rPBP3) within a period of 10 to 15 days when exposed to β-lactams.^46^

A number of amino acid substitutions and combinations have been identified among our strains. However, it remains unclear whether all these affect the resistance level. It is possible that certain amino acid substitutions may serve to restore proper protein function as bacteria attempt to compensate for the effects of certain amino acid substitutions (e.g. N526K), which, although leading to β-lactam resistance, interfere with correct PBP3 function. For a more comprehensive understanding of the issue, it would be optimal to clone individual amino acid substitutions and their combinations into a WT strain; however, this procedure is beyond the capabilities of our laboratory.

Our work has primarily focused on determining the detection capability of disc diffusion and MIC to recognize *H. influenzae* strains with amino acid substitutions in PBP3 protein, and determining the categorizing agreement of the results by the aforementioned methods. The findings indicate that, despite the impact of the selection of strains with only amino acid substitutions, the screening method to identify the non-enzymatic mechanism of resistance, as recommended by EUCAST, using the penicillin disc is applicable in the majority of cases. However, in certain instances, screening with an ampicillin disc or, in exceptional cases, a cefuroxime disc may be a more accurate predictor of strains with modified PBP3. The use of the kappa statistic also demonstrated a markedly low level of interpretative agreement between the methods employed, thereby indicating that laboratories would be better advised to test MICs. It is important to note that the phenotypic (genotypic) detection of rPBP strains cannot be considered a substitute for standard susceptibility testing. Rather, it should be regarded as an adjunct to a more comprehensive understanding of the issue of β-lactam resistance in *H. influenzae*.

## Supplementary Material

dkaf023_Supplementary_Data

## Supplementary data

Table S1 is available as Supplementary data at *JAC* Online.
