# Supplementary material for: Amino acid substitutions in PBP3 in Haemophilus influenzae strains, their phenotypic detection and impact on resistance to β-lactams
Source: J Antimicrob Chemother. 2025 Feb 3;80(4):980–7. doi: 10.1093/jac/dkaf023 (PMC11962375; doi:10.1093/jac/dkaf023)
Supplement: dkaf023_Supplementary_Data [file dkaf023_supplementary_data.docx]

**Table S1**. Categorical agreement (agreement of interpretation in % between MIC and disk diffusion methods), detection value for rPBP3 (how much the combination of the method and the antibiotic is able to detect rPBP3 strains, in %), very major error (i.e., susceptible by disk diffusion and resistant by broth microdilution MIC, in %) and major error (i.e., resistant by disk diffusion and susceptible by broth microdilution MIC, in %) for combination of all detected AA combinations and MIC values for ampicillin and cefuroxime. For penicillin, CA, VME and ME values are given in relation to ampicillin MIC.

| **AA substitution (combination)** | **Penicillin** | **Ampicillin** | | | | | **Cefuroxime** | | | | | **Penicillin disk/ampicillin MIC** | | |
| --- | --- | --- | --- | --- | --- | --- | --- | --- | --- | --- | --- | --- | --- | --- |
| **MIC (mg/L)** | DV (%) disk | CA (%) | VME (%) | ME (%) | DV (%) disk | DV (%) MIC | CA (%) | VME (%) | ME (%) | DV (%) disk | DV (%)  MIC | CA (%) | VME (%) | ME (%) |
| **D350N** | 100 | 0.0 | 0.0 | 100 | 100 | 0.0 | - | - | - | - | - | 0.0 | 0.0 | 100 |
| 0.25 |  | 0.0 |  |  |  |  |  |  |  |  |  | 0.0 |  |  |
| **T532S** | 0.0 | 0.0 | 0.0 | 100 | 100 | 0.0 | 0.0 | 0.0 | 100 | 100 | 0.0 | 100 | 0.0 | 0.0 |
| 0.25 |  | 0.0 |  |  |  |  |  |  |  |  |  | 100 |  |  |
| 2 |  |  |  |  |  |  | 0.0 |  |  |  |  |  |  |  |
| **D350N, A437S** | 0.0 | 0.0 | 0.0 | 100 | 100 | 0.0 | - | - | - | - | - | 100 | 0.0 | 0.0 |
| 0.125 |  | 0.0 |  |  |  |  |  |  |  |  |  | 100 |  |  |
| **A502V, R517H** | 33.3 | 0.0 | 33.3 | 66.7 | 66.7 | 33.3 | 0.0 | 100 | 0.0 | 0.0 | 100 | 66.7 | 33.3 | 33.3 |
| 1 |  | 0.0 |  |  |  |  |  |  |  |  |  | 50.0 |  |  |
| 2 |  | 0.0 |  |  |  |  |  |  |  |  |  | 100 |  |  |
| 4 |  |  |  |  |  |  | 0.0 |  |  |  |  |  |  |  |
| **A437S, A502V, N526K** | 0.0 | 0.0 | 0.0 | 100 | 100 | 0.0 | 0 | 0.0 | 100 | 100 | 0.0 | 100 | 0.0 | 0.0 |
| 1 |  | 0.0 |  |  |  |  |  |  |  |  |  | 100 |  |  |
| 2 |  |  |  |  |  |  | 0 |  |  |  |  |  |  |  |
| **D350N, A502V, N526K** | 100 | 0.0 | 0.0 | 100 | 100 | 0.0 | - | - | - | - | - | 0.0 | 0.0 | 100 |
| 1 |  | 0.0 |  |  |  |  |  |  |  |  |  | 0.0 |  |  |
| **D350N, M377I, G490E, R501C, A502V, N526K** | 0.0 | 0.0 | 0.0 | 100 | 100 | 0.0 | 100 | 0.0 | 0.0 | 100 | 100 | 100 | 0.0 | 0.0 |
| 1 |  | 0.0 |  |  |  |  |  |  |  |  |  | 100 |  |  |
| 4 |  |  |  |  |  |  | 100 |  |  |  |  |  |  |  |
| **D350N, S357N, M377I, S385T, R517H, T532S** | 100 | 33.3 | 0.0 | 66.7 | 100 | 33.3 | 91.7 | 0.0 | 8.3 | 91.7 | 83.3 | 33.3 | 0 | 66.7 |
| 1 |  | 0.0 |  |  |  |  |  |  |  |  |  | 0.0 |  |  |
| 2 |  | 100 |  |  |  |  | 50.0 |  |  |  |  | 100 |  |  |
| 4 |  |  |  |  |  |  | 100 |  |  |  |  |  |  |  |
| 8 |  |  |  |  |  |  | 100 |  |  |  |  |  |  |  |
| >8 |  |  |  |  |  |  | 100 |  |  |  |  |  |  |  |
| **I449V, N526K** | 100 | 40.0 | 0.0 | 60.0 | 100 | 40.0 | 100 | 0.0 | 0.0 | 100 | 100 | 40.0 | 0.0 | 60.0 |
| 1 |  | 0.0 |  |  |  |  |  |  |  |  |  | 0 |  |  |
| 2 |  | 100 |  |  |  |  |  |  |  |  |  | 100 |  |  |
| 4 |  |  |  |  |  |  | 100 |  |  |  |  |  |  |  |
| 8 |  |  |  |  |  |  | 100 |  |  |  |  |  |  |  |
| **D350N, A502T, N526K** | 100 | 40.0 | 0.0 | 60.0 | 100 | 40.0 | 100 | 0.0 | 0.0 | 100 | 100 | 60.0 | 0.0 | 40.0 |
| 1 |  | 0.0 |  |  |  |  |  |  |  |  |  | 33.3 |  |  |
| 2 |  | 100 |  |  |  |  |  |  |  |  |  | 100 |  |  |
| 4 |  |  |  |  |  |  | 100 |  |  |  |  |  |  |  |
| 8 |  |  |  |  |  |  | 100 |  |  |  |  |  |  |  |
| >8 |  |  |  |  |  |  | 100 |  |  |  |  |  |  |  |
| **N526K** | 100 | 50.0 | 0.0 | 50.0 | 100 | 50.0 | 75.0 | 0.0 | 25.0 | 100 | 75.0 | 50.0 | 0.0 | 50.0 |
| 1 |  | 0.0 |  |  |  |  |  |  |  |  |  | 0.0 |  |  |
| 2 |  | 100 |  |  |  |  | 0.0 |  |  |  |  | 100 |  |  |
| 4 |  |  |  |  |  |  | 100 |  |  |  |  |  |  |  |
| 8 |  |  |  |  |  |  | 100 |  |  |  |  |  |  |  |
| >8 |  |  |  |  |  |  | 100 |  |  |  |  |  |  |  |
| **A502V, N526K** | 75.0 | 62.5 | 0.0 | 37.5 | 75.0 | 62.5 | 50 | 16.7 | 33.3 | 83.3 | 66.7 | 62.5 | 0.0 | 37.5 |
| 1 |  | 40.0 |  |  |  |  |  |  |  |  |  | 40.0 |  |  |
| 2 |  | 100 |  |  |  |  | 0.0 |  |  |  |  | 100 |  |  |
| 4 |  |  |  |  |  |  | 75.0 |  |  |  |  |  |  |  |
| **D350N, M377I, G490E, A502V, N526K** | 90.9 | 63.6 | 0.0 | 36.4 | 100 | 63.6 | 90.9 | 0.0 | 9.1 | 90.1 | 81.8 | 72.7 | 0.0 | 27.3 |
| 1 |  | 0.0 |  |  |  |  |  |  |  |  |  | 25 |  |  |
| 2 |  | 100 |  |  |  |  | 50.0 |  |  |  |  | 100 |  |  |
| 4 |  |  |  |  |  |  | 100 |  |  |  |  |  |  |  |
| 8 |  |  |  |  |  |  | 100 |  |  |  |  |  |  |  |
| >8 |  |  |  |  |  |  | 100 |  |  |  |  |  |  |  |
| **M377I, I449V, N526K** | 100 | 66.7 | 0.0 | 33.3 | 100 | 66.7 | 66.7 | 0.0 | 33.3 | 66.7 | 100 | 66.7 | 0.0 | 33.3 |
| 1 |  | 0.0 |  |  |  |  |  |  |  |  |  | 0 |  |  |
| 2 |  | 100 |  |  |  |  |  |  |  |  |  | 100 |  |  |
| 4 |  |  |  |  |  |  | 0.0 |  |  |  |  |  |  |  |
| 8 |  |  |  |  |  |  | 100 |  |  |  |  |  |  |  |
| >8 |  |  |  |  |  |  | 100 |  |  |  |  |  |  |  |
| **D350N, G490E, N526K, A530S** | 95.2 | 81.0 | 0.0 | 19.0 | 90.5 | 71.4 | 84.2 | 5.3 | 10.5 | 89.5 | 84.2 | 76.2 | 0.0 | 23.8 |
| 1 |  | 33.3 |  |  |  |  |  |  |  |  |  | 16.7 |  |  |
| 2 |  | 100 |  |  |  |  | 33.3 |  |  |  |  | 100 |  |  |
| 4 |  | 100 |  |  |  |  | 80.0 |  |  |  |  | 100 |  |  |
| 8 |  |  |  |  |  |  | 100 |  |  |  |  |  |  |  |
| >8 |  |  |  |  |  |  | 100 |  |  |  |  |  |  |  |
| **N526K, A530S** | 100 | 83.3 | 0.0 | 0.0 | 83.3 | 83.3 | 83.3 | 0.0 | 16.7 | 83.3 | 66.7 | 83.3 | 0.0 | 16.7 |
| 1 |  | 0.0 |  |  |  |  | 100 |  |  |  |  | 0.0 |  |  |
| 2 |  | 100 |  |  |  |  | 0.0 |  |  |  |  | 100 |  |  |
| 4 |  |  |  |  |  |  | 100 |  |  |  |  |  |  |  |
| 8 |  |  |  |  |  |  | 100 |  |  |  |  |  |  |  |
| >8 |  |  |  |  |  |  | 100 |  |  |  |  |  |  |  |
| **D350N, M377I, A502V, N526K** | 100 | 87.1 | 0.0 | 12.9 | 100 | 87.1 | 86.2 | 0.0 | 13.8 | 96.6 | 82.8 | 87.1 | 0.0 | 12.9 |
| 1 |  | 0.0 |  |  |  |  |  |  |  |  |  | 0.0 |  |  |
| 2 |  | 100 |  |  |  |  | 20.0 |  |  |  |  | 100 |  |  |
| 4 |  |  |  |  |  |  | 100 |  |  |  |  |  |  |  |
| 8 |  |  |  |  |  |  | 100 |  |  |  |  |  |  |  |
| >8 |  |  |  |  |  |  | 100 |  |  |  |  |  |  |  |
| **D350N, N526K** | 50.0 | 100 | 0.0 | 0.0 | 50.0 | 50.0 | 100 | 0.0 | 0.0 | 100 | 100 | 0.0 | 50.0 | 50.0 |
| 0.5 |  | 100 |  |  |  |  |  |  |  |  |  | 0.0 |  |  |
| 2 |  | 100 |  |  |  |  |  |  |  |  |  | 0.0 |  |  |
| >8 |  |  |  |  |  |  | 100 |  |  |  |  |  |  |  |
| **D350N, N526K, A530S** | 100 | 100 | 0.0 | 0.0 | 100 | 100 | 100 | 0.0 | 0.0 | 100 | 100 | 100 | 0.0 | 0.0 |
| >8 |  | 100 |  |  |  |  | 100 |  |  |  |  | 100 |  |  |
| **G490E, A502V, N526K** | 100 | 100 | 0.0 | 0.0 | 100 | 100 | 100 | 0.0 | 0.0 | 100 | 100 | 100 | 0.0 | 0.0 |
| 2 |  | 100 |  |  |  |  |  |  |  |  |  | 100 |  |  |
| 8 |  |  |  |  |  |  | 100 |  |  |  |  |  |  |  |
| **G490E, N526K, A530S** | 100 | 100 | 0.0 | 0.0 | 100 | 100 | 100 | 0.0 | 0.0 | 100 | 100 | 100 | 0.0 | 0.0 |
| 2 |  | 100 |  |  |  |  |  |  |  |  |  | 100 |  |  |
| 8 |  |  |  |  |  |  | 100 |  |  |  |  |  |  |  |
| **D350N, S357N, A502V, N526K** | 100 | 100 | 0.0 | 0.0 | 100 | 100 | 100 | 0.0 | 0.0 | 100 | 100 | 100 | 0.0 | 0.0 |
| 4 |  | 100 |  |  |  |  | 100 |  |  |  |  |  |  |  |
| **G490E, A437S, A502V, N526K** | 100 | 100 | 0.0 | 0.0 | 0.0 | 0.0 | 100 | 0.0 | 0.0 | 0.0 | 0.0 | 0.0 | 0.0 | 100 |
| 0.5 |  | 100 |  |  |  |  | 100 |  |  |  |  | 0.0 |  |  |
| **S406G, P408S, V418A, A437S, V461I, I519L, N526K, A530S** | 100 | 100 | 0.0 | 0.0 | 100 | 100 | 0.0 | 0.0 | 100 | 100 | 0.0 | 100 | 0 | 0 |
| 2 |  | 100 |  |  |  |  | 0.0 |  |  |  |  | 100 |  |  |
| **D350N, S357N, M377I, S385T, L389F, N526K** | 100 | 100 | 0.0 | 0.0 | 100 | 100 | 100 | 0.0 | 0.0 | 100 | 100 | 100 | 0.0 | 0.0 |
| 4 |  | 100 |  |  |  |  |  |  |  |  |  | 100 |  |  |
| 8 |  | 100 |  |  |  |  |  |  |  |  |  | 100 |  |  |
| >8 |  |  |  |  |  |  | 100 |  |  |  |  | 100 |  |  |
| **D350N, S357N, M377I, S385T, L389F, A502V, N526K** | 100 | 100 | 0.0 | 0.0 | 100 | 100 | 100 | 0.0 | 0.0 | 100 | 100 | 100 | 0.0 | 0.0 |
| >8 |  | 100 |  |  |  |  | 100 |  |  |  |  | 100 |  |  |
| **D350N, S357N, M377I, S385T, L389F, I449V, N526K** | 100 | 100 | 0.0 | 0.0 | 100 | 100 | 100 | 0.0 | 0.0 | 100 | 100 | 100 | 0.0 | 0.0 |
| 4 |  | 100 |  |  |  |  |  |  |  |  |  | 100 |  |  |
| >8 |  |  |  |  |  |  | 100 |  |  |  |  |  |  |  |
| **D350N, S357N, M377I, S385T, L389F, R517H, T532S** | 100 | 100 | 0.0 | 0.0 | 100 | 100 | 100 | 0.0 | 0.0 | 100 | 100 | 100 | 0.0 | 0.0 |
| 4 |  | 100 |  |  |  |  |  |  |  |  |  | 100 |  |  |
| 8 |  | 100 |  |  |  |  | 100 |  |  |  |  | 100 |  |  |
| >8 |  | 100 |  |  |  |  | 100 |  |  |  |  | 100 |  |  |
| **D350N, S357N, M377I, S385T, L389F, G490E, N526K, A530S** | 100 | 100 | 0.0 | 0.0 | 100 | 100 | 100 | 0.0 | 0.0 | 100 | 100 | 100 | 0.0 | 0.0 |
| 8 |  | 100 |  |  |  |  |  |  |  |  |  | 100 |  |  |
| >8 |  |  |  |  |  |  | 100 |  |  |  |  |  |  |  |
| **M377I, S385T, L389F, R517H, T532S** | 100 | 100 | 0.0 | 0.0 | 100 | 100 | 100 | 0.0 | 0.0 | 100 | 100 | 100 | 0.0 | 0.0 |
| 4 |  | 100 |  |  |  |  |  |  |  |  |  | 100 |  |  |
| >8 |  |  |  |  |  |  | 100 |  |  |  |  |  |  |  |

CA - Categorical agreement; DV – Detection value for AA substitutions in PBP3; VME - Very major error; ME - Major error.
